# Intravenous Infusion of Autoserum-Expanded Autologous Mesenchymal Stem Cells in Patients With Chronic Brain Injury: Protocol for a Phase 2 Trial

**DOI:** 10.2196/37898

**Published:** 2022-07-06

**Authors:** Shinichi Oka, Tomohiro Yamaki, Masanori Sasaki, Ryo Ukai, Mitsuhiro Takemura, Takahiro Yokoyama, Yuko Kataoka-Sasaki, Rie Onodera, Yoichi M Ito, Shigeki Kobayashi, Jeffery D Kocsis, Yasuo Iwadate, Osamu Honmou

**Affiliations:** 1 Department of Neural Regenerative Medicine Research Institute for Frontier Medicine Sapporo Medical University School of Medicine Sapporo Japan; 2 Department of Advanced Regenerative Therapeutics Sapporo Medical University School of Medicine Sapporo Japan; 3 Division of Neurosurgery Rehabilitation Center for Traumatic Apallics Chiba National Agency for Automotive Safety and Victims' Aid Chiba Japan; 4 Department of Neurology Yale University School of Medicine New Haven, CT United States; 5 Data Science Center Institute of Health Science Innovation for Medical Care Hokkaido University Hospital Sapporo Japan; 6 Department of Neuroscience Yale University School of Medicine New Haven, CT United States; 7 Department of Neurological Surgery Chiba University Graduate School of Medicine Chiba Japan

**Keywords:** autologous mesenchymal stem cells, chronic brain injury, cell therapy, intravenous delivery, stem cells, rehabilitation, brain injury, brain, motor vehicle accident, falls, hypoxia

## Abstract

**Background:**

Brain injuries resulting from motor vehicle accidents and falls, as well as hypoxic insults and other conditions, are one of the leading causes of disability and death in the world. Current treatments are limited but include continuous rehabilitation, especially for chronic brain injury. Recent studies have demonstrated that the intravenous infusion of mesenchymal stem cells (MSCs) has therapeutic efficacy for several neurological diseases, including stroke and spinal cord injury.

**Objective:**

The objective of our investigator-initiated clinical trial is to assess the safety and potential efficacy of the intravenous infusion of autoserum-expanded autologous MSCs for patients with chronic brain injury.

**Methods:**

The (phase 2) trial will be a single-arm, open-label trial with the primary objective of confirming the safety and efficacy of autoserum-expanded autologous MSCs (STR-01; produced under good manufacturing practices) when administered to patients with chronic brain injury. The estimated number of enrolled participants is 6 to 20 patients with a modified Rankin Scale grade of 3 to 5. The assessment of safety and the proportion of cases in which the modified Rankin Scale grade improves by 1 point or more at 180 days after the injection of STR-01 will be performed after MSC infusion.

**Results:**

We received approval for our clinical trial from the Japanese Pharmaceuticals and Medical Devices Agency on December 12, 2017. The trial will be completed on June 11, 2023. The registration term is 5 years. The recruitment of the patients for this trial started on April 20, 2018, at Sapporo Medical University Hospital in Japan.

**Conclusions:**

Our phase 2 study will aim to address the safety and efficacy of the intravenous infusion of MSCs for patients with chronic brain injury. The use of STR-01 has been performed for patients with cerebral infarction and spinal cord injury, providing encouraging results. The potential therapeutic efficacy of the systemic administration of autoserum-expanded autologous MSCs for chronic brain injury should be evaluated, given its safety and promising results for stroke and spinal cord injury.

**Trial Registration:**

Japan Medical Association Center for Clinical Trials JMA-IIA00333; https://tinyurl.com/nzkdfnbc

**International Registered Report Identifier (IRRID):**

DERR1-10.2196/37898

## Introduction

### Background

A *brain injury* is defined as an alteration in brain function or other evidence of brain pathology caused by trauma, including trauma resulting from motor vehicle accidents, falls, hypoxic insults, infections, and other conditions. Survivors—not only in severe cases but also in moderate or mild brain injury cases—experience the significant burdens of physical and neuropsychological disabilities. These disabilities disrupt the lives of patients and their families and result in substantial health care and social costs [1]. Chronic histopathological changes, such as cell death, axonal injury, vascular damage, and inflammation, have long-term persistence in brain injury survivors [2]. Thus, it is important to develop a novel approach to treating chronic brain injury.

The intravenous infusion of mesenchymal stem cells (MSCs) has shown therapeutic efficacy in experimental animal models of neurological diseases and injuries, including cerebral ischemia [3-12], spinal cord injury (SCI) [13-16], chronic epilepsy [17], and peripheral nerve injury [18-20]. The suggested therapeutic mechanisms of MSCs from animal studies include the secretion of neurotrophic factors that can provide neuroprotection [14,17,21], neovascularization [22,23], the restoration of the blood-brain barrier [13,24], the regeneration of axonal injury [13,25], remyelination [13], synaptogenesis [12,25], induced neural plasticity [12,25], and remote effects [15]. These therapeutic mechanisms may have beneficial effects on chronic brain injury as well. We also conducted clinical studies in which the intravenous infusion of autologous MSCs in patients with stroke [26,27] and SCI [28] was performed, and we showed its safety and improvements in neurologic symptoms. Thus, we hypothesize that the intravenous infusion of MSCs may have therapeutic efficacy for patients with chronic brain injury.

### Objectives

The objectives of the proposed clinical trial include evaluating the safety and efficacy of intravenously infused autologous MSCs that are expanded with autosera in patients with chronic brain injury.

## Methods

### Study Design

**Figure 1:**
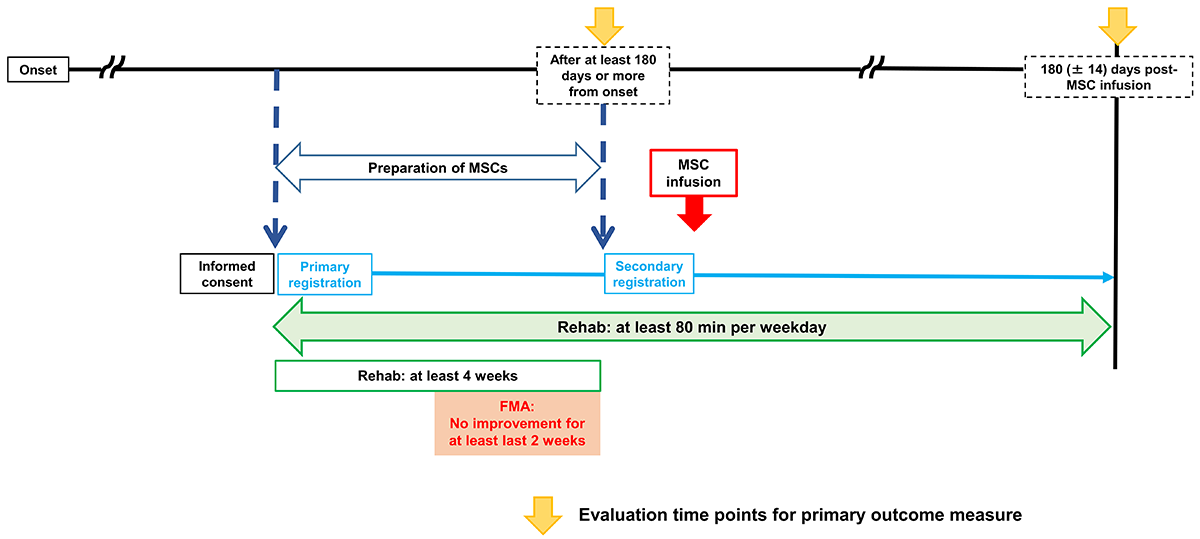
Clinical protocol. FMA: Fugl-Meyer assessment; MSC: mesenchymal stem cell.

The (phase 2) trial will be a single-arm, open-label trial. The outline of the clinical protocol is shown in Figure 1. We will infuse the MSCs at least 180 days after onset. This trial will be carried out at Sapporo Medical University Hospital, Japan. The study protocol was based on advice provided by the Pharmaceuticals and Medical Devices Agency (PMDA) in Japan.

We propose to provide an extensive rehabilitation protocol to all participants prior to MSC infusion in order to exclude the potential effects of rehabilitation alone and evaluate MSC-specific effects. Briefly, all patients with chronic brain injury will receive formal rehabilitation (at least 80 min/weekday for 4 weeks) and continue formal rehabilitation until they show no further improvements in Fugl-Meyer assessment (FMA) scores for the last 2 weeks prior to MSC infusion. Thus, we expect to evaluate the therapeutic effects of MSC infusion in addition to the effects of rehabilitation therapy. We will evaluate FMA scores approximately every week. The clinical data will be collected by at least 1 physical therapist and at least 1 Japanese board-certified neurosurgeon at Sapporo Medical University Hospital. If the patients require more than 2 weeks to reach an FMA score plateau, we will continue formal rehabilitation until they show no further improvements for the next 2 weeks.

For cell preparation, peripheral blood draws from each patient for autoserum and bone marrow collection will be performed. The autologous MSCs will be cultured in autologous sera, and the autologous human MSCs (called *STR-01*) will be manufactured in a cell processing center at Sapporo Medical University. STR-01 will be prepared under good manufacturing practice conditions by personnel, who have received formal good manufacturing practice training, within a facility with highly controlled temperature, room air, pressure, and environmental conditions and then cryopreserved at −150 °C until its use, as previously described [28]. On the day of infusion, cryopreserved units will be thawed at the bedside in a 37 °C water bath and will be administered with saline to each patient for approximately 60 minutes. The total number of cells in the STR-01 product is based on our previous study (0.5 × 10^8^ to 2 × 10^8^ cells per patient) [27]. Hospital treatment, including rehabilitation for a target of 80 min/weekday, will be performed for 180 (±14) post-MSC infusion days, after which final outcome measures will be evaluated.

### Sample Size

We will recruit 10 people (minimum: 6; maximum: 20) with a modified Rankin Scale (mRS) grade of 3 to 5. The focus of our trial is to establish safety and potential efficacy. However, it should be noted that if the proportion of cases in which the mRS grade improves by 1 point or more exceeds 10%, the trial will provide significant clinical benefit. To detect a difference by using a *Z*-test with continuity correction, a minimum of 6 people is required. Thus, the target sample size of people with an mRS grade of 3 to 5 was determined to be 10 (minimum: 6; maximum: 10), under the consideration of dropout.

### Ethics Approval

This study protocol was approved by the institutional review board (IRB) at Sapporo Medical University Hospital (approval number: 29-15). Changes to the protocol will require IRB and Japanese PMDA approval. The trial will be conducted in accordance with the ethical principles of the Declaration of Helsinki, the Japanese Pharmaceutical Affairs Law, and good clinical practice. Trial investigators will obtain written informed consent from each participant before study inclusion. If the participants do not have the ability to write, written informed consent will be obtained from a legal representative alone. Participants will be free to withdraw from the study at any time.

### Consent for Publication

The results from our study will be presented in peer-reviewed publications and meetings without identifying data. Written consent for publication will be obtained from all participants in the study.

### Eligibility Criteria

Since MSCs will be collected from the participants (autologous MSCs) in our study, we must start the MSC cultures after primary registration in order to initiate the collection of peripheral blood and bone marrow when the protocol therapy starts. Afterward, we must confirm that the MSC product passed the shipping standards before infusion. Thus, we must perform a secondary registration of the participants. Therefore, case registration will require 2 steps in the trial; we will register the participants before blood and bone marrow collections (first registration) and before the infusion of MSCs (second registration).

### Inclusion Criteria at the Time of the First Registration

For the first registration, the inclusion criteria will be as follows: (1) a brain injury oth.er than stroke diagnosed via magnetic resonance imaging (MRI), computed tomography, 3D computed tomography angiography (3D-CTA), or angiography (including a suspected brain injury other than stroke); (2) a classification of grade 3 to 5 on the mRS; (3) patients aged 20 to 80 years; (4) patients whose rehabilitation can be performed for at least 80 minutes per weekday; and (5) written informed consent, which will be obtained as much as possible from the participants. If a participant does not have ability to write, written informed consent will be obtained from a legal representative alone.

### Exclusion Criteria at the Time of the First Registration

For the first registration, the exclusion criteria will be as follows:

1. Severe disturbance of consciousness (a Japan Coma Scale score of between 200 and 300)
2. Severe contracture, deformity, or calcification of a joint
3. Diagnosed with hepatitis B, hepatitis C, or syphilis via initial screening
4. Pancytopenia (a white blood cell concentration of <2000 cells/µL, hemoglobin concentration of <10.0 g/dl, or platelet concentration of <100,000 platelets/µL)
5. MRI (or computed tomography) scan revealing a severe asymptomatic lesion or white matter lesion
6. MRI scan revealing multiple and severe instances of microbleeding or hemosiderosis in the whole brain
7. Head and neck magnetic resonance angiography (or 3D-CTA or angiography) scan revealing ≥70% stenosis of main cerebral arteries and cervical carotid arteries even after the revascularization (except for complete occlusion) or dissection of an artery
8. Head and neck magnetic resonance angiography (or 3D-CTA or angiography) scan revealing severe arteriosclerotic change or severe calcification
9. Moyamoya disease, cerebral aneurysm, and other vascular malformations with a high risk of rupture or cerebral embolism
10. Uncontrollable hypertension with therapy prior to infusion (systolic pressure: >140 mm Hg; diastolic pressure: >90 mm Hg)
11. Past history of neoplasms (except complete response); severe diseases of the blood and blood-forming organs; certain disorders involving the immune mechanism; severe mental and behavioral disorders; severe diseases of the nervous system; and severe congenital malformations, deformations, and chromosomal abnormalities
12. Past history of penicillin and streptomycin allergy or other severe allergy (shock or anaphylactic symptoms)
13. Poor general condition due to endocrine, nutritional, and metabolic diseases; uncontrollable mental disorders; diseases of the nervous system (refractory epilepsy), diseases of the circulatory system (uncontrollable and refractory heart failure, moderate or severe valvular heart disorder, uncontrollable and refractory atrial fibrillation, refractory atrial and ventricular thrombi, a history of ischemic heart disease with percutaneous coronary intervention within the past 12 months, and serious arrhythmia); diseases of the respiratory system; diseases of the digestive system; diseases of the musculoskeletal system and connective tissue; diseases of the genitourinary system (dialysis); injury; poisoning; and certain other consequences of external causes
14. Participation in another clinical trial related to any organ or a past history of cellular therapy
15. Pregnant or possibly pregnant women, nursing women, women who plan to be pregnant during the study period, or male patients who want their partners to get pregnant
16. Other patients who are judged by investigators holding a medical license as inappropriate for the study

### Inclusion Criteria at the Time of the Second Registration

For the second registration, the inclusion criteria will be as follows: (1) ≥180 days after the onset of a brain injury other than stroke; (2) patients who, after rehabilitation for at least 80 minutes per weekday or as much as possible over the past 1 month or more, showed no improvement in FMA score over the last 2 weeks (definition of *improvement in FMA score*: improvement by 1 point or more, as shown by the total FMA score); (3) a classification of grade 3 to 5 on the mRS; and (4) patients who are ready for the infusion of STR-01 that satisfies the specifications of the acceptance criteria.

### Exclusion Criteria at the Time of the Second Registration

For the second registration, the exclusion criteria will be as follows:

1. Severe disturbance of consciousness (Japan Coma Scale score of between 200 and 300)
2. Diagnosed with hepatitis B, hepatitis C, HIV, human T-lymphotropic virus 1 infection, syphilis, or human parvovirus B19 infection via detailed examination
3. More than 70% stenosis of main cerebral arteries and cervical carotid and vertebral arteries even after the revascularization (except for complete occlusion and a healed dissecting artery) or dissection of an artery
4. Severe arteriosclerotic change and calcification
5. Moyamoya disease, cerebral aneurysm, or other vascular malformations with a high risk of rupture or cerebral embolism
6. Uncontrollable hypertension with therapy prior to infusion
7. Ischemic heart disease (more than 75% stenosis of coronary arteries)
8. Cardiac shunt malformation (ventricular septal defect or arterial septal defect)
9. Possible large thrombus, as determined via laboratory examination
10. Neoplasms (except complete response); severe diseases of the blood and blood-forming organs; certain disorders involving the immune mechanism; severe mental and behavioral disorders; severe diseases of the nervous system; and severe congenital malformations, deformations, and chromosomal abnormalities
11. Penicillin and streptomycin allergy and other severe allergy (shock or anaphylactic symptoms)
12. Poor general condition due to endocrine, nutritional, and metabolic diseases; uncontrollable mental disorders; diseases of the nervous system (refractory epilepsy), diseases of the circulatory system (uncontrollable and refractory heart failure, moderate or severe valvular heart disorder, uncontrollable and refractory atrial fibrillation, refractory atrial and ventricular thrombi, a history of ischemic heart disease and percutaneous coronary intervention within the past 12 months, and serious arrhythmia); diseases of the respiratory system; diseases of the digestive system; diseases of the musculoskeletal system and connective tissue; diseases of the genitourinary system (dialysis); injury; poisoning; and certain other consequences of external causes
13. Pregnant women, nursing women, those who plan to be pregnant during the study period, or male patients who want their partners to get pregnant
14. Other patients who are judged by investigators holding a medical license as inappropriate for the study

### Outcome Measures

Outcome measures will be performed by more than 2 Japanese board-certified neurosurgeons at Sapporo Medical University Hospital**.**

#### Primary Outcome

The primary outcome is the proportion of cases in which the mRS grade improves by 1 point or more between 180 (±14) days after the injection of STR-01 and just before injection (−14 to 0 days).

#### Secondary Outcomes

The following are the secondary outcomes: (1) the rate of all adverse events during the whole study period, (2) the differences in FMA scores (each item score and total score) between 180 (±14) days after the injection of STR-01 and just before injection (−14 to 0 days), (3) the differences in National Institutes of Health Stroke Scale scores (each item score and total score) between 180 (±14) days after the injection of STR-01 and just before injection (−14 to 0 days), and (4) the differences in Functional Independence Measure scores (each item score and total score) between 180 (±14) days after the injection of STR-01 and just before injection (−14 to 0 days).

### Statistical Analysis

The plan of analysis for study data will be performed by a biomedical statistician (YMI), and statistical analyses will be performed by using JMP 11.1 for Windows (SAS Institute Inc). The details are described in our statistical analysis plan and are described briefly in the following subsections.

#### Primary Outcome Measure

The valid target population is patients with an mRS grade of 3 to 5 before MSC infusion. We assume that the proportion of cases without MSC infusion in which the mRS grade improves by 1 point or more will be 0.1%. We will perform a Z-test with continuity correction at 180 (±14) days after MSC infusion.

#### Secondary Outcome Measure

We will estimate the proportion of cases with at least 1 level of improvement in mRS grade and the 95% CI by using the scoring method at 180 (±14) days after MSC infusion. We will also estimate the mean changes in FMA, National Institutes of Health Stroke Scale, and Functional Independence Measure scores from immediately before (−14 to 0 days) MSC infusion to 180 (±14) days after MSC infusion and the 95% CIs by using the Wald method.

### Provisions for Posttrial Care

The participants of the trial will follow standard clinical procedures during the study; thus, there will be no specific posttrial care. As the participants are the patients within Japan’s National Health Insurance system, postcare will be provided through the National Health Insurance schemes, if necessary. Clinical research insurance for studies will also be covered.

### Access to Data

All investigators will have access to the trial data.

### Data Management

All participant trial data will be entered into the electronic data capture system hosted at the Translational Research Center for Medical Innovation (Kobe, Japan). The data collected will be deidentified by using unique study code numbers. To maintain the privacy of the participants, any reports of individual data will only consist of clinical data without any names, addresses, or identifying information. This complies with the university’s IRB guidelines. All patient-related information and data that are generated will be maintained on a secure server. Data monitoring will comply with the university’s policies, its guidelines, and the data management plan that was approved for the study. Data will be audited at an appropriate period by the EPS Corporation (Tokyo, Japan). At the completion of the study, the results will be submitted for publication in a peer-reviewed journal and presented at national and international conferences.

## Results

We received approval for our clinical trial from the Japanese PMDA on December 12, 2017. The trial will be completed on June 11, 2023. The registration term is 5 years. The recruitment of the patients for this trial started on April 20, 2018, at Sapporo Medical University Hospital in Japan.

## Discussion

The specific objective of our trial is to evaluate the safety and potential therapeutic efficacy of the intravenous infusion of autoserum-expanded autologous MSCs for patients with chronic brain injury. The patients who enrolled in our study with chronic brain injury and decreased neural function due mainly to brain trauma will receive an intravenous infusion of autologous MSCs that are expanded in autosera. A brain injury presents serious health and socioeconomic burdens that the development of an effective therapy could alleviate.

Various treatments have been developed that focus mainly on the acute phase, including neurorestorative, anti-inflammatory, and neuroprotective agents. However, no established medical therapies that promote effective therapeutic efficacy have been made, especially for the chronic phase. Therefore, a novel therapy that promotes recovery from brain damage after a chronic brain injury should be developed [29].

We previously reported on the safety, feasibility, and potential therapeutic efficacy of the intravenous infusion of autoserum-expanded autologous MSCs for patients with cerebral infarction [27] and subacute SCI [28]. The intravenous infusion of MSCs derived from bone marrow improves functional outcomes in experimental animal models of stroke [4-7,9-11], SCI [13,14,16,24,30,31], neonatal hypoxic ischemia [12], chronic epilepsy [17], cerebral small vessel disease [8,32], amyotrophic lateral sclerosis [33,34], and peripheral nerve injury [18,21]. Although the mechanisms underlying these beneficial effects have not been fully elucidated, potential mechanisms include neuroprotection and immunomodulation [14], the induction of axonal sprouting [13], remyelination [13], the restoration of the blood-brain and blood–spinal cord barriers [13,24], and the enhancement of remote gene expression responses [15].

We reported that infused MSCs facilitate neural plasticity in experimental models of neonatal [12] and adult [4,5] cerebral ischemia. Since brain injuries in the chronic phase are heterogenous injuries that are underpinned by numerous complex and interrelated pathophysiological conditions [35], it is conceivable that the enhanced neural plasticity resulting from MSC injection promotes structural rewiring, which might contribute to functional improvement in chronic state of neural diseases. In addition, there are other multimodal and orchestrated mechanisms, as shown in previous studies [3-13,15-18,25,28,32-34,36-38]. Given these considerations of the potential therapeutic effects of MSCs in a number of neurological disorders, we planned a clinical trial for chronic brain injury.

The purpose of our study is to address the safety and potential therapeutic efficacy of the intravenous infusion of autologous MSCs based on the primary outcome measures. If the intravenous infusion of autologous MSCs shows possible therapeutic efficacy and is shown to be safe without any major adverse effects, this approach could be successfully translated to a larger controlled and blinded clinical study in the future. The data from our study will be used to develop a new clinical protocol for any future, larger, definitive evaluation trials.

In conclusion, due to the promising therapeutic effects of autoserum-expanded autologous MSCs for stroke [27] and SCI [28], this approach should be evaluated for chronic brain injury, which shares many of the histopathological conditions that have been seen in patients with stroke and SCI. We thus believe that this cell therapy approach for patients chronic brain injury could have a significant impact and warrants evaluation in the near future [39].

## Abbreviations

3D-CTA: 3D computed tomography angiography
FMA: Fugl-Meyer assessment
IRB: institutional review board
MRI: magnetic resonance imaging
mRS: modified Rankin Scale
MSC: mesenchymal stem cell
PMDA: Pharmaceuticals and Medical Devices Agency
SCI: spinal cord injury
